# Barriers of Physician-Patient Relationships in Professionalism: A Qualitative Study

**DOI:** 10.30476/JAMP.2022.94010.1563

**Published:** 2022-07-01

**Authors:** MOHAMMAD HASAN KESHAVARZI, SAEEDE SAFAIE, SEYED ALI AKBAR FAGHIHI, SOOLMAZ ZARE

**Affiliations:** 1 Clinical Education Research Center, Faculty of Medicine, Shiraz University of Medical Sciences, Shiraz Iran; 2 Shiraz University of Medical Sciences, Shiraz, Iran

**Keywords:** Communication, Barriers, Professionalism

## Abstract

**Introduction:**

Undoubtedly, there are several obstacles in the path of medical professionalism. This study examines these obstacles in the relationship between physician and patient. Therefore, this study was conducted to explore the barriers of physician-patient relationships in professionalism based on physician experiences.

**Methods:**

It was a qualitative study with a conventional content analysis approach. Our participants were 14 patients and 11 physicians. The sampling method was purposive, and data was collected through semi-structured interviews and field notes. Interviews continued until data saturation.

**Results:**

Findings of the study regarding barriers of patient-physician relationship were classified into five main categories: misperception of the physician's identity, unprofessional behavior of the physician, physician’s sense of self-superiority, patients' cultural differences, and lack of supportive services in the health care system.

**Conclusion:**

The results of this study showed that the communication challenges between physician and patient were not limited to the physicians' education. Part of these challenges depends on the patients and their culture. Another part of these challenges is directly related to the medical and management system structure.

## Introduction

Communication skills are an essential part of life skills defined as communicating effectively and efficiently with others leading to responses ( 1 ). This relationship is formed when the physician and the patient consciously tend to accept each other ( 2 ). An effective physician-patient relationship increases the patients' confidence and willingness to communicate ( 3 ). Several studies have confirmed the influential role of patient-physician communication in treatment outcomes. According to the available reports, establishing a proper relationship between the physician and patient can improve the patient’s satisfaction with the treatment process and attract participation, leading to faster recovery and better outcomes ( 4 ). A good physician-patient relationship positively affects the patients' health and recovery ( 5 ). Stavropolu (2011), in a study entitled the "Non-Compliance with the Treatment and the Physician-Patient Relationship" which was conducted across Europe, concluded that the physician-patient relationship plays a significant role in the patients̕ decision-making to accept and follow the treatment recommendations, and a good relationship can predict the patients̕ follow-up outcomes ( 6 ). Warner, in a study, showed that the "Physicians believe that their skills are such that the patients are not in a position to judge how they are treated and how they act because they are unprofessional and cannot make judgments about the physicians' performance on a non-professional basis. These assessments are also about the interaction between two different and conflicting sets of norms ( 7 ). In a research paper, Delghandi investigated the causes regarding the propagation of the patients' complaints to the physicians and stated that: "According to the medical experts, in most cases, physicians did not commit the negligence, and most of the complaints were about the inappropriate interaction between the physician and the patient and the patient's lack of proper justification before treatment” ( 8 ). A literature review showed that although most studies have emphasized the critical effect of the physician-patient relationship during treatment and recovery, limited research has been done in Iran on this critical issue. As one of the most up-to-date and vital issues in social research globally, it has received less attention in Iran ( 6 ).

There are many obstacles to establishing strong relationships between physicians and patients in medical activities. Undoubtedly, in medical professionalism, identifying some of these obstacles will improve the physician-patient relationship and improve the quality of medical care ( 9 ). This study was conducted to explore barriers of physician-patient relationships in professionalism based on physician experiences.

## Methods

This is a qualitative study using conventional content analysis. 

### Data Collection

Interviewees were selected from patients and physicians in outpatient centers in Shiraz. The sampling method was purposeful. Inclusion criterion was willingness to retell clinical environment experiences. Sampling was continued until data saturation. Twenty-five people, including 11 physicians and 14 patients, participated in the study. Interviews with patients who had previously made an appointment with a physician were coordinated. Patients who were interested could participate in the interview. The interview took place in a quiet room.

Semi-structured interviews were used to collect the data. The interviews lasted at least 30 minutes. All interviews were recorded and then written word for word and analyzed. The interview guide included some general questions about the topic. For example, for physician: How would you describe your job experience in the clinic? What problems did you encounter during the physical examination? What factors influence your relationship with the patient? For the patient: What experience have you had with your doctor? How does the doctor interact with you? What is the barrier between you and the doctor? The interviews were listened to several times until a general idea was obtained. The ambiguities were resolved by rechecking the transcribed data with the participants after the interview.

### Data analysis

*Graneheim and Lundman's* method was used to analyzed the data ( 10 ). This is the way to analyze interviews. In this method, the researcher first reads the text several times. Then, coding is done. After that, the sub-categories and categories are obtained. Codes were extracted and classified based on semantic units derived from the participants' descriptions. Attempts were made to have the highest homogeneity within the categories and the most heterogeneity. 

### Accuracy and Strength of the Data

Lincoln and Guba's criteria were used to check the accuracy of the data ( 11 ). These criteria in qualitative research are known as credibility, dependability, confirmability, and transferability. To ensure the credibility of the data, being in the field for a long time, peer check, and establishment of appropriate communication with participants were considered. To be sure about the dependability of the data, an expert in qualitative studies who was not involved in the study checked the data. About the conformability, a complete description of the steps of the study was described. Data source triangulation was used. To determine transferability, we asked an expert to recheck the study steps and extract the codes. 

### Ethical Considerations

Regarding ethical considerations, all participants were first explained about the purpose and method of the study, and then a written informed consent form was completed by them. The ethical code of research is IR.SUMS.REC.1398.1137.

## Results

Twenty-five interviewees, including 11 physicians and 14 patients, 16 females, and 9 males, participate in this study. Interviews were transcribed and analyzed, which resulted in 174 codes that were classified into 19 subcategories and 5 main categories: misperception of the physician's identity, unprofessional behavior of the physician, physician’s sense of self-superiority, patients' cultural differences, and lack of supportive services in the health care system (Table 1).

**Table 1:** Subcategories, categories, and theme of barriers of physician-patient relationships in professionalism

Theme	Categories	Subcategories
Communication barriers	Misperception of the physician's identity	Secretary interaction with the patient
		Physician's work experience
		The effect of media on the patient
	Unprofessional behavior of the physician	Ignoring the patient's question
		favoritism
		Insufficient empathy with the patient
		Lack of non-verbal communication between physician and patient
		Creating fear and anxiety in the patient
	Physician sense of self-superiority	Doctor's inattention to the patient's complaint
		Disrespect for patient time
		Making patients feel being indebted to doctors
	Patients' cultural differences	Unusual patient expectations
		Lack of proper understanding of the examination and diagnosis process
		Ignoring the doctor's recommendations
		Poor interaction with the doctor
	Lack of supportive services in the health care system	Disregarding patients’ autonomy
		Improper clinical environment
		Excessive number of patients
		Informing the patient inappropriately

### Misperception of the physician's identity

This category includes the following three subcategories: the secretary interaction with the patient, physician's work experience, and the effect of media on the patient. 

### • Secretary interaction with the patient

The secretary is the first person in the treatment team to communicate with the patient before the physician. As a result, his attitude will influence the patient's mental image of the physician. For example, one participant stated:

*"I tell the secretary that my mother has high blood pressure. Let her go to the physician sooner. "She says, "Well, It’s none of my business."*

*"Why does she tell me what to do? I'm sure that the secretary tells the physician to ignore us. Thanks God, they are nothing."*

### • Physician's work experience

The behavior and working experience of the physician directly influence the patient's perception, as one of the participants stated:

*"Well, before I enter the physician's room, some factors influence me. For example, the secretary's behavior, patients' speeches, and even the good and bad reputation of the physician, their behavior, and even their skill all play a role in my opinion about doctors. However, the first contact of the physician is significant. It really can change any misunderstanding."*

### • Effect of media on the patient

Meanwhile, the press, media, and even advertisements have had a significant effect on creating an image away from reality, creating a level of irrational expectation in the patients, which is a deterrent factor to communication. For example, one of the participants said:

*"Radio, television, and the press are hurting the communications. They think they are doing a good job. If they continue to hurt the communications, some physicians do not learn; some issues are not observed; this hurts the patients. They change the level of their expectations. As a result, they do not have the logical expectations from the physician."*

### Unprofessional behavior of the physician

It involves the experiences of the participants believing that the physician has behaved in an unprofessional manner, which is expressed in five subcategories, including ignoring the patient's question, favoritism, insufficient empathy with the patient, lack of non-verbal communication between physician and patient, and creating fear and anxiety in the patient.

### • Ignoring the patient's question

Most of the participants mentioned it in the same way; one of the participants stated that:

*".... What makes you think you are not happy with your physician? Not only I get no results from treatment, but also, it bothers me when a physician ignores me, disrespects me, and does not listen to me. When I ask a question, he continues with his job order, not even looking at me and finally not answering the question."*

### • Favoritism

Both the patient and the physician mentioned favoritism and preferential treatment as the factor which leads to miscommunication. In this regard, one of the physicians stated:

*A physician stated: "Patients who are referred to a doctor by a friend create a double emotional burden and responsibility in performing their duties."*

*A patient said: "I was referred to the doctor by a friend. The doctor examined my condition thoroughly and treated me with respect, which I think he did so because a friend introduced me."*

### • Insufficient empathy with the patient

When the patients feel they are not understood by the physicians, they emphasize the lack of empathy by the physicians. For example, one of the participants stated:

*"They don't spend enough time with the patient and have very little empathy, and have no mutual understanding of the patient. Some time, we went to the physician with my mother; I expressed my mother's problem, but the doctor ignored me and wrote down what was on her mind. She was only there and had no precision or empathy about the problem, to declare that she understood what we said ".*

### • Lack of non-verbal communication between physician and patient

When a physician uses only his or her words to communicate with patients despite using words with body language, it is a clear example that non-verbal communication is not being considered an integral part of effective communication. Most patients and even doctors pointed out the lack of proper use of non-verbal communication; one of the doctors stated:

*"It is enough for this relationship to be damaged somewhere, for example, in critical issues, disruption in face-to-face communication with the patient. Alternatively, it is enough that the doctor looks or pays attention to anything instead of the patient. This condition indicates the fragility of the relationship between doctor and patient."*

As one of the patients said:

*"First, I say hello when the physician looks at me; then I start to express my condition; the physician's behavior is so important to me."*

### • Creating fear and anxiety in the patient

If the patient is not adequately informed about her disease, it results in fear and anxiety caused by the ambiguous condition, which negatively impacts his/her relationship with the doctor. One of the participants pointed out:

*" I had gallstone surgery on March 11th. The doctor examined me and said ’the pain you were having was not essential and that there is no need to surgery right now. However, you will be hospitalized for the next two or three days to follow up the cause of the pain. After an hour, they took me to the operating room in a rush. I was amazed and wanted to ask the doctor the reason for the immediate operation. In the operating room, I asked one of the staff ‘why are you operating on me.’ Before anesthesia, I insisted on seeing the doctor, but unfortunately, I was anesthetized and operated on before seeing a doctor, and this situation is embarrassing."*

### Physician’s sense of self-superiority

Another major category derived from data analysis was "physician’s sense of self-superiority," which included three subcategories: "Doctor's inattention to the patient's complaint," "disrespect for the patient’s time," and "making patients feel being indebted to doctors." 

### • Doctor's inattention to the patient's complaint

It is about ignoring the patient by the doctor without justifying the patient, which will lead to an effective lack of communication. 

*"We asked the doctor to postpone the training class after examining the patients due to our tiredness and that of our patients, most of whom were children. However, he did not pay any attention." said one participant.*

### • Disrespect for patient’s time

The lack of respect for the patients̕ time is disrespectful to the patient. One of the participants stated:

*"Well, I didn't expect it to be this way at all. I don't think they had any respect for their patients or their patients' time. They should have paid attention to this issue, not just their own plans or and time."*

### • Making patients feel being indebted to doctors

This physician's verbal and non-verbal approach shows that he/she does not have the required humility to empathize with the patient to communicate effectively; this plays a significant role in weakening the physician's relationship with the patient. 

In this regard, one of the participants stated: *"He treated us with egotism and made us wait until 8 p.m.; it was awful, and they think they are God."*

### Patients' cultural differences

The "patients' cultural differences" reflect the beliefs, customs, and behaviors rooted in one's culture. Because people from different cultures refer to educational hospitals, their cultural diversity prevents effective communication between physicians and patients. According to the participants' experiences, four subcategories were found: "Unusual patient expectations," "Lack of proper understanding of the examination and diagnosis process", "Ignoring the doctor's recommendations", and "Poor interaction with the doctor."

### • Unusual patient expectations

One of the unreasonable expectations that the patient shows in his/her relationship with the physician is expressed in the following statement by one of our participants: *"Some patients without prior coordination expect to meet the doctor very soon without considering the rights and priorities of other patients or expect to get consultation about the medical condition of several members of their family at the time of their examination.”*

### • Lack of Proper Understanding of the Examination and Diagnosis Process

Patients' lack of knowledge about the process of examination, diagnosis, treatment, and even follow-up is one of the obstacles to communication between patients and physicians. In this regard, one of the physicians stated: *"Patients think and expect that I should always be available. Some patients talk a lot, some have little information, and some do not understand what I explain for them."*

### • Ignoring the doctor's recommendations

When the patients do not follow the physician's prescriptions and recommendations or do not take clinical tests, it can result in dissatisfaction with the physicians.

 For instance, one physician said: *"There are some patients who ignore the follow-ups. For example, they don't do the prescribed clinical tests at all or at the right time and don't take the medications properly in cases of time and dosage."*

### • Poor interaction with the doctor

The interaction between the physician and patient is a multifaceted process in which achieving the desired result requires a mutual and effective interaction between them. 

One physician said: "I learned how to be a physician, but as a patient, most of them don't know how to behave." In another interview, one of the participants emphasized: *"The situation will be much better if the patients are more aware of their rights and process of their cure. (for example, what they should ask the doctor, what should the doctor do, checking blood pressure, or taking a medical history, doing a physical exam,etc)."*

### Lack of supportive services in the health care system

Another main category that emerged in this study is "lack of supportive services in the health care system," which can result in many problems, one of which is miscommunication between patients and physicians. It includes four subcategories: "Disregarding the patients' autonomy", "Improper clinical environment", "Excessive number of patients", "Informing the patient inappropriately."

### • Disregarding patients' autonomy

The patient has the right to choose his physician and treatment procedure. In this study, both groups of participants somehow pointed out that the patient did not have the right to choose. One of the physicians said: *"The public health care system is very ineffective; the patients have no autonomy. In reality, they use the obligatory services because of their financial problems and the higher cost of the same services in the private health care system. If I visit 70 patients a day, I am not proud at all, or it is not amazing; I know that at least half of them did not choose me on cognition and knowledge."*

One participating patient said: *"The doctor did not explain my condition fully and I didn't understand what were my other options for treatment; he just prescribed the medicines."*

### • Improper clinical environment

It refers to the clinical environment that can afford the basic rights of the patients. In this regard, one of the physicians said: *“The ventilation is not ok, the secretary behavior is always disrespectful, the nurses and the staff don’t do their duties as well, everywhere is crowded, and mismanagement is crystal clear in all layers of the health care system. Waiting time for any kind of care is so long. Anyway, I mean they run a healthy person to an outrage, crazy one. By the way, how can we expect to communicate clearly with each other in such a miserable context?”*

### • Excessive number of patients

The higher the number of patients, the lower the quality of communication between the physician and the patient, thus weakening the process of effective communication between the physician and the patient; as one of the patient said:

"Regardless of what we say, he decides and writes a prescription. Of course, in the public health care system, the price of care is lower; on the other hand, to compensate for their expenditure, they make the physicians work faster. Lack of time and too many patients are all the story of miscommunication between the patient and doctor."

### • Informing the patient inappropriately

Many of the patient's issues, such as accommodation or insurance condition, or getting aware of the absence of a physician, etc., can be eliminated by giving patients timely and adequate information. However, inappropriately informing the patient is one of the communication barriers as announced in the form of complaints about the health care system by many participants. In this regard, one of the participants said:

*"We live in another city; we drive 4 hours to get here. About 2 months ago, I set our appointment with the physician for today, and now they say he is on vacation. I told them that why they didn't inform us; instead of apologies, they rudely shouted out loud that it was not their duty to inform us."*

## Discussion

The present study investigated the barriers to establishing optimal communication between the physician and patient. Based on the findings of this study, communication barriers are a set of factors preventing the formation of effective communication between the physician and patient or reducing the quality of this communication, including misperception of the physician's identity, unprofessional behavior of the physician, physician sense of self-superiority, patients' cultural differences, and lack of supportive services in the health care system. 

### Misperception of the physician's identity

According to the results of this study, this concept indicates that giving awareness and knowledge to the patients leads to a negative effect on the quality of communication between the physician and patient. In this regard, Sun et al. showed that the negative news hurt the inpatient's understanding of physician-patient relationships ( 12 ). It seems that the factors, such as the behavior of the secretary with the patient, physician's work experience and team work skills, and the effect of the media on the patient,play a significant role in this regard.

### Unprofessional behavior of the physician

Based on the participants' perceptions and experiences, they were not paying attention to the patient's question, favoritism, lack of empathy with the patient, lack of respect for the patients̕ time, and lack of non-verbal communication between the physician and the patient all cause anxiety in patients. Patients expect the physicians to fully listen to what they want to say, ask a variety of questions to find out how they feel about the disease, feel empathy and being understood by the physician, and also interact with them to alleviate their sickness and pain ( 13 - 15 ).

### Physician’s sense of self-superiority

The concept of physicians' sense of self-superiority includes the physician's disregard for the patient's complaints, disrespect for the patient's time, and obligation by the physician. In a study, Robert Ratbarton et al. (2016) investigated the relationship between the physicians̕ humility, physician-patient relationship, and the patients̕ health. Their results showed a relationship between the physicians̕ humility and effective communication between the physician and patient, so that the humble physicians were more successful in interacting with their patients than the arrogant ones ( 16 , 17 ). The physician was shown to be a key indicator for evaluating the quality of services. Paying attention to this factor plays an essential role in the patients̕ satisfaction ( 18 , 19 )

### Patients' cultural differences

This concept refers to the patient's improper expectations, lack of understanding of the examination and diagnosis process, failure to follow the physician's advice, and weakness in interaction with the physician. Insufficient patients̕ educational level reduces the patients̕ awareness and trust in the physician-patient relationship. To emphasize the role of cultural weakness of the patients, Zheng in a study showed that the interaction between the physicians and patients was significantly compromised when the cultural perspectives of the participants were ignored. As a result of confusion, uncertainty was one of the issues raised by the patients ( 20 , 21 ).

### Lack of supportive services in the health care system

This is another concept that refers to the lack of choice by the patient, unfavorable treatment environment, high number of patients, and weakness in informing the patient. In line with this concept, Chandra et al. (2018), in a review paper entitled "Trust and Communication in the Physician-Patient Relationship," suggested that a patient-centered approach with practical communication skills and self-confidence leads to better management and ultimately the patients̕ satisfaction. Continuation of this process is associated with better treatment, health outcomes, and understanding of health care quality ( 22 ). Giving appropriate information to a patient is crucial, but choosing the right information strategy is non-negligible ( 23 ).

Thus, it seems that the patients should have the right to choose their physician. They should be encouraged to consider quality as the most important reason for choosing a physician because the conscious selection of a physician by the patient will lead to competition between the physicians. As a result, physicians become more forced to improve the quality of their services to satisfy the patients and meet their logical needs and expectations ( 24 ). On the other hand, the high number of patients can be an effective structural attenuator in patient-physician communication. In this regard, many studies have shown that following the increase in the number of patients receiving health care services in the educational hospital, the medical errors of the physicians and staff also increase, which is also related to stress, anxiety, and burnout ( 25 , 26 ); moreover, lack of time to clearly explain about the patients’ condition results in serious complication in patient-doctor interactions ( 27 , 28 ), so it is recommended that more studies should be conducted to clarify this issue.

We had time limitations in our study. Because some of the interviewees were patients, they were not very willing to cooperate after the interview. They did not answer their phone if we had questions after the interview. In order to remove this limitation, we made an attempt to ask all the questions and ambiguities of the interviewee during the interview.

## Conclusion

This study showed that the communication challenges between the physician and patient are not limited to physicians' education. Part of these challenges depends on the patients and their culture. Another part of these challenges is directly related to the structure of the medical and managerial system.

The lack of quality in the communication process between the physician and patient will have many consequences, such as patient safety issues, adverse effects on the public opinion, reduced empathy between the physician-patient, and challenges of medical professionalism. Lack of optimal treatment increases the complaints from the physicians. Therefore, holding a program about physician-patient communication skills should be seriously considered in the form of textbooks in the training courses for the students and workshops and continuous training of the physicians. 

## Acknowledgement

The authors would like to thank all of the people who took part in this study.

## Authors' contribution

M.H.K, S.S, S.A.A.F, S.Z contributed to the conception and design of the work; the acquisition, analysis, or interpretation of data for the work. All Authors contributed in drafting and revising the manuscript critically for important intellectualcontent. All authors have read and approved the final manuscript and agree to be accountable for all aspects of the work in ensuring that questions related to the accuracy or integrity of any part of the work are appropriately investigated and resolved.

## Conflict of Interest:

None declared.
